# The association between ambient temperature and mortality in South Africa: A time-series analysis

**DOI:** 10.1016/j.envres.2017.11.001

**Published:** 2018-02

**Authors:** Noah Scovronick, Francesco Sera, Fiorella Acquaotta, Diego Garzena, Simona Fratianni, Caradee Y. Wright, Antonio Gasparrini

**Affiliations:** a Woodrow Wilson School, Princeton University, Princeton, NJ 08544, USA; b Department of Social and Environmental Health Research, London School of Hygiene and Tropical Medicine, London WC1H 9SH, UK; c Department of Earth Sciences, University of Turin, Turin 10124, Italy; d Environment and Health Research Unit, South African Medical Research Council and Department of Geography, Geoinformatics and Meteorology, University of Pretoria, Pretoria 0001, South Africa

**Keywords:** Temperature, Weather, Climate, Mortality, South Africa, Cardiovascular, Respiratory

## Abstract

**Background:**

There is an extensive literature describing temperature-mortality associations in developed regions, but research from developing countries, and Africa in particular, is limited.

**Methods:**

We conducted a time-series analysis using daily temperature data and a national dataset of all 8.8 million recorded deaths in South Africa between 1997 and 2013. Mortality and temperature data were linked at the district municipality level and relationships were estimated with a distributed lag non-linear model with 21 days of lag, and pooled in a multivariate meta-analysis.

**Results:**

We found an association between daily maximum temperature and mortality. The relative risk for all-age all-cause mortality on very cold and hot days (1st and 99th percentile of the temperature distribution) was 1.14 (1.10,1.17) and 1.06 (1.03,1.09), respectively, when compared to the minimum mortality temperature. This “U” shaped relationship was evident for every age and cause group investigated, except among 25–44 year olds. The strongest associations were in the youngest (< 5) and oldest (> 64) age groups and for cardiorespiratory causes. Heat effects occurred immediately after exposure but diminished quickly whereas cold effects were delayed but persistent. Overall, 3.4% of deaths (~ 290,000) in South Africa were attributable to non-optimum temperatures over the study period. We also present results for the 52 district municipalities individually.

**Conclusions:**

An assessment of the largest-ever dataset for analyzing temperature-mortality associations in (South) Africa indicates mortality burdens associated with cold and heat, and identifies the young and elderly as particularly vulnerable.

## Highlights

• Country-wide epidemiology study of temperature-mortality association in S. Africa.
• Strongest associations were in children/elderly and from cardiorespiratory causes.
• Total attributable mortality was 3.4%, mostly from cold (3.0%) rather than heat (0.4%).
• Attributable mortality of 3.4% is on the low end of what is reported in other areas.
• Results can help estimate future burdens from climate change and inform adaptation.

## 1 Introduction

There is an extensive literature describing temperature-mortality associations in developed regions, but research from developing countries, and Africa in particular, is limited (Basu, 2009, Benmarhnia et al., 2015, Gasparrini et al., 2015b, Ryti et al., 2016). Furthermore, the few studies from Africa report results for only one or a few cities and over relatively short time periods (Azongo et al., 2012, Diboulo et al., 2012, Egondi et al., 2012, McMichael et al., 2008, Wichmann, 2017).

There are several reasons that the relationship between ambient temperature and mortality in Africa may differ when compared to wealthier regions. Populations often have distinct mortality profiles (cause/age of death) and age distributions, while climatic factors may also differ. Additionally, large segments of the population live in dwellings that do not adequately protect against heat and cold (Makaka and Meyer, 2006, Scovronick and Armstrong, 2012, United Nations Human Settlements Programme, 2011). All of these factors are known or putative modifiers of the effect of temperature on mortality (Basu, 2009, Benmarhnia et al., 2015, Ryti et al., 2016, Scovronick and Armstrong, 2012).

The urgency to better understand temperature-health relationships in developing regions is heightened when considering the near-term opportunities for intervention; rapid rates of economic development and demographic change, combined with explicit government programs aimed at infrastructure upgrading and poverty alleviation may all affect vulnerability to ambient temperature. Examples in South Africa include government programs to help provide ~ 1.5 million homes to low-income households over the coming years as well as household energy and water supply projects (Department of Energy (Republic of South Africa), 2015, Department of Human Settlements (Republic of South Africa), 2016, Department of Water and Sanitation (Republic of South Africa), 2014).

The shortage of information on how temperature affects health in South Africa is of extra concern in the context of climate change. Mean warming has increased at least 50% faster in South Africa compared to the global average and (like for much of the continent) this trend is expected to continue (Niang, 2014, Ziervogel et al., 2014). As a result, there is strong consensus that adaptation will be key to protect populations in Africa from future climate impacts and that early action is needed (Niang, 2014, Schaeffer, 2013). However, the Intergovernmental Panel on Climate Change, along with independent researchers and the South African government have all pointed to a lack of research on climate- and weather-health relationships as a barrier to climate-informed decision-making (Government of the Republic of South Africa, 2011, Niang, 2014; Smith et al., 2014; Wright et al., 2014; Ziervogel et al., 2014).

Accordingly, in this study we analyze the association of temperature and mortality in South Africa using a national dataset that includes all 8.8 million recorded deaths between 1997 and 2013. This is the first study to employ such a large-scale dataset from anywhere in Africa, and to our knowledge no comparable study exists from any country at a similar level of economic development.

## 2 Methods

We conducted a time-series regression analysis of the temperature-mortality relationship in South Africa using a national mortality dataset and two independent sources of temperature data.

### 2.1 Mortality dataset

**Fig. 1:**
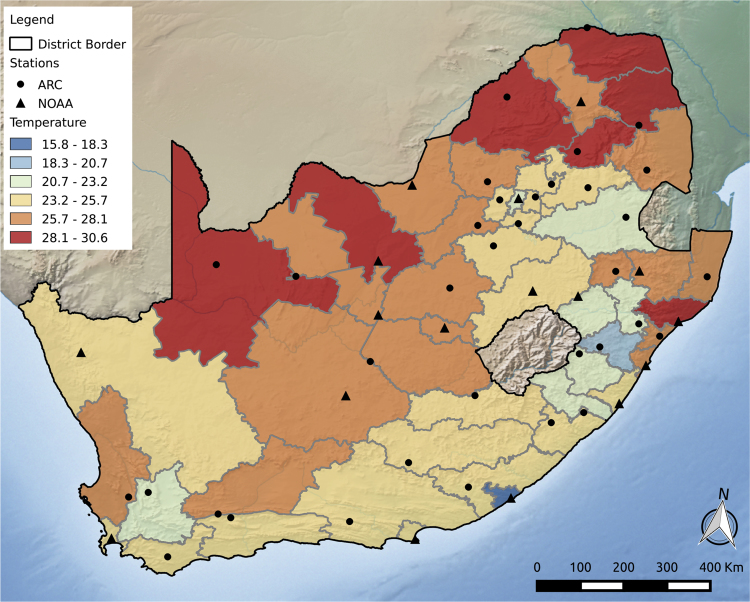
Mean maximum temperature for each district, location of the weather monitoring station, and source of the data. ARC = Agricultural Research Council (South Africa), NOAA = National Oceanographic and Atmospheric Administration (USA).

We obtained a dataset of all recorded deaths (n = 8,814,625) in South Africa from 1997 to 2013, inclusive (17 years). The information is from the country's civil registration system, the only national source of mortality statistics. The dataset was provided by Statistics South Africa, which estimates that death registration for adults is ~ 89% complete early in the study period, rising to ~ 94% by the end (completeness of child records has not been reported) (Statistics South Africa, Mortality and causes of death in South Africa, 2011, Statistics South Africa, Mortality and causes of death in South Africa, 2013). Anonymized individual data reported where each death occurred at the level of the district municipality (there are 52 in South Africa and we refer to them hereafter as “districts”). District sizes range from relatively small urban areas to much larger areas located in the more unpopulated regions of the country (Fig. 1). In addition to death district, individual data included cause and age of death. Data on district of residence was not available.

After dropping records for stillbirths (n = 226,593), deaths with incomplete district information (n = 82,154) and with an incorrect (nonsensical) date of death (n = 49), the final all-cause, all-age dataset consisted of 8,509,130 records. The number of all-age all-cause deaths for each district can be found in Table S1 of the Supplementary material.

### 2.2 Temperature dataset

We obtained daily minimum and maximum temperature data from two sources: the National Oceanographic and Atmospheric Association (NOAA) of the United States and South Africa's Agricultural Research Council. The NOAA dataset included 63 daily series covering a subset of 30 districts, while the Agricultural Research Council dataset included 50 series, one for each district except the City of Johannesburg and Nelson Mandela Bay. There was no overlap in measurement points (stations) between the two sources.

We carried out a three-step quality control procedure on all temperature series from both sources to exclude values resulting from either erroneous transcription or instrument malfunction (Aguilar et al., 2003, Alexander and Herold, 2016, Perkins et al., 2012, Zhang et al., 2011). First, we removed records where the maximum temperature was lower than the minimum. Second, we compared every series with corresponding data from two or more nearby weather stations, selected for proximity and data correlation with the reference series (Milewska and Vincent, 2016, Venema et al., 2012). If the reference series recorded an outlier (values above/below the 90th/10th percentiles), that outlier was removed if the difference with the comparison series was ± two root root-mean-square errors. The root-mean-square error between the reference and comparison series was calculated daily. Additionally, we removed all duplicate sets – defined as periods with at least five consecutive days recording the same temperature. And third, for series exhibiting a break in recording or where metadata indicated a change or relocation of the instrument, the series was tested for homogeneity and corrected if necessary (Fortin et al., 2016, Wang and Feng, 2010, Wang, 2008a, Wang, 2008b, Wang et al., 2010, Wang et al., 2007). Only one district (Buffalo City) required an adjustment.

After the quality control procedure, we assembled a final composite dataset consisting of one representative station for each district (Fig. 1), selected for the length of the series and fewest missing data points. In the first year of the study period, there was temperature data available for 29 districts, rising to cover all 52 by 2007 (Fig. S1). For each series in the final dataset, we reconstructed missing data if the data gap was less than or equal to six days in length, using information from the nearby comparison stations (Acquaotta et al., 2009, Acquaotta et al., 2016, Eischeid et al., 2000).

Overall, seven percent of values were reconstructed for the daily maximum and 12% for the daily minimum temperature. Due to the higher number of missing values and because improper instrument management generally has a greater impact on minimum temperature recordings (Acquaotta et al., 2015, Caussinus and Mestre, 2004, Nigrelli et al., 2015, Rangwala and Miller, 2012, Trewin, 2010, Vincent et al., 2009), all subsequent analyses in this paper use the daily maximum temperature.

### 2.3 Statistical approach

We applied a two-stage time-series modeling strategy, described in several recent methodological papers and previously applied in both multi-city and multi-country contexts, thus allowing for consistency and comparability between studies (Gasparrini, 2011, Gasparrini et al., 2012, Gasparrini et al., 2015a, Gasparrini et al., 2016, Gasparrini et al., 2015b). We briefly describe the modeling stages below and also refer readers to these prior publications for more details (Gasparrini, 2011, Gasparrini et al., 2012, Gasparrini et al., 2015a, Gasparrini et al., 2016, Gasparrini et al., 2015b).

In the first stage, we applied standard time-series quasi-Poisson regression models separately for each district to derive estimates of location-specific temperature-mortality associations. For this step, we modeled the exposure-response association with a natural cubic spline with three internal knots at the 10th, 75th and 90th percentiles of location-specific temperature and the lag-response association using a natural cubic spline with an intercept and three knots equally spaced on the log scale. We controlled for season and trend with a natural cubic spline with eight degrees of freedom per year and also for day of the week.

In the second stage, we reduced the association in two dimensions: first to the overall temperature-mortality association, cumulating the risk over a 21-day lag period to account for the delayed effects of cold and for potential short-term mortality displacement; then to the lag-response associations corresponding to the 99th and 1st percentiles using the district-specific minimum mortality temperature (MMT) as a reference. We pooled the estimated location-specific estimates using a multivariate meta-analytical model, controlling for location-specific average (maximum) temperature and temperature range. The fitted meta-analytical model was used to derive the best-linear unbiased prediction of the overall cumulative exposure-response association in each district.

We quantified the total attributable number of all-cause deaths associated with non-optimum temperatures, which is given by the sum of the contributions from all days in the series, and its ratio with the total number of deaths provides the total attributable fraction. We calculated the components attributable to cold and heat by summing the subsets corresponding to days with temperatures below or above the MMT using the district-specific temperature distributions. To explore the effect of extreme cold and heat specifically, we also report attributable mortality due to temperatures below and above the 2.5th and 97.5th temperature percentiles, respectively.

We calculated empirical confidence intervals for the exposure-response curves using Monte Carlo simulations assuming a multivariate normal distribution of the best linear unbiased predictions of the reduced coefficients. Confidence intervals for the MMTs were determined using an approximate parametric bootstrap estimator (Tobías et al., 2016).

The modeling strategy described above was selected from several potential approaches and therefore we conducted sensitivity analyses on key modeling choices including the lag period, the degrees of freedom used for seasonal control and in the lag-response association, and the type of spline and knot location for the exposure-response association.

All analyses were conducted with the R software packages *dlnm* and *mvmeta*, which have been used extensively and are documented in detail elsewhere (Gasparrini, 2011, Gasparrini et al., 2012, Gasparrini et al., 2015b).

## 3 Results

The mean daily maximum temperature for South Africa as a whole during the study period was 25.3 °C. District-level means ranged from 15.8 °C (Buffalo City) to 30.6 °C (Vhembe) (Fig. 1, Table S1), with an interquartile range between 21.7 °C and 29.2 °C. In total, 89% (n = 7,576,674) of all deaths in the database had matching temperature data and could therefore be included in the temperature-mortality analyses (Table 1).

**Table 1:** Deaths analyzed and results from pooled analyses including minimum mortality temperature percentiles (MMP) and relative risks at select temperatures compared to the minimum.

**Age/cause group**	**Deaths**^a^		**MMP**	**Relative risk**	
	**Total**	**Included**^b^		**1**st **percentile**	**99**th **percentile**
All-cause, all-age	8,509,130	7,576,674	84 (56,89)	1.14 (1.10,1.17)	1.06 (1.03, 1.09)
Cardiovascular^c^	1,299,688	1,154,896	80 (64,89)	1.33 (1.24,1.42)	1.08 (1.02, 1.14)
Respiratory^d^	1,046,914	931,556	84 (60,90)	1.16 (1.07,1.25)	1.10 (1.03,1.18)
Other causes	6,162,528	5,490,222	84 (27,90)	1.09 (1.06,1.12)	1.05 (1.01,1.09)
< 5 years	800,268	700,088	26 (1,44)	1.08 (0.99,1.18)	1.24 (1.15,1.34)
5–24 years	623,687	553,088	19 (9,99)	1.10 (1.00,1.20)	1.10 (1.00,1.20)
25–44 years	2,665,115	2,382,762	95 (1,99)	1.02 (0.97,1.06)	1.00 (0.97,1.04)
45–64 years	2110,984	1,889,622	81 (47,99)	1.16 (1.09,1.22)	1.04 (0.99,1.08)
65+ years	2,265,979	2,016,041	85 (74,88)	1.34 (1.28,1.41)	1.13 (1.07,1.20)

MMP: Minimum mortality temperature percentile.

a Excludes stillbirths, deaths without location information and nonsensical dates of death.

b Deaths were included for all days with temperature data.

c ICD-10: I00-I99.

d ICD-10: J00-J99.

**Fig. 2:**
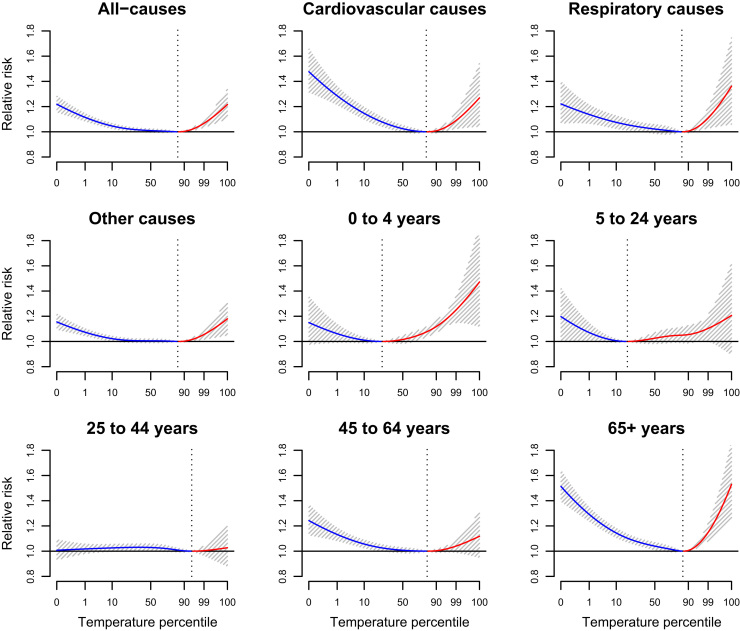
Pooled overall relative risks of mortality associated with maximum daily temperature cumulated over a 21-day lag period for different cause and age groups. Dotted vertical line show the minimum mortality temperature percentile.

Fig. 2 displays pooled results for the overall cumulative exposure-response curves by age and cause groupings. The all-age all-cause curve shows elevated mortality on both sides of the temperature distribution, with relative risks of up to ~ 20% at the extreme ends when compared to the minimum mortality temperature. Heat and cold effects are evident for all the age and cause groupings, with the exception of 25–44 year olds. The steepest curves are found in the youngest and (in particular) oldest age groups. Cardiovascular and respiratory diseases also exhibited higher risks from cold and heat, respectively.

The best-linear unbiased predictions of the overall cumulative exposure response associations for all-age all-cause mortality in each of the 52 districts are presented in Fig. S2. Like for the pooled results, most districts show evidence of a ‘U’ shaped curve, although several do not have clear cold and/or heat effects, the latter including some of the most populous urban districts (e.g. City of Cape Town and City of Johannesburg). There is evidence of moderate heterogeneity across districts (Cochran Q p-value < 0.001, I^2^ = 41.9%).

**Fig. 3:**
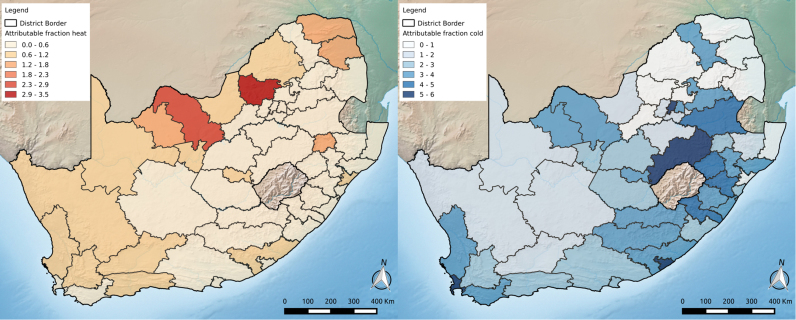
Attributable fraction for heat and cold by district. Exact values available in Table S1.

The proportion of deaths nationally attributable to low and high temperatures combined was 3.0% and 0.4%, respectively, for a total attributable fraction from non-optimum temperatures of 3.4%. The district range was 1–6% (Fig. 3, Table S1). Only a small percentage (~ 13%) of the attributable mortality was due to extreme temperatures (Table S2).

The high proportion of total temperature-attributable deaths related to cold is a function of the cold slope and the relatively high minimum mortality temperature (Table 1). High minimum mortality temperatures were also evident in the other age- and cause-groups, with the exception of the youngest groups (Table 1). A total mortality burden of 3.4% is equivalent to ~ 290,000 deaths, or an average of ~ 17,000 per year, over the study period.

**Fig. 4:**
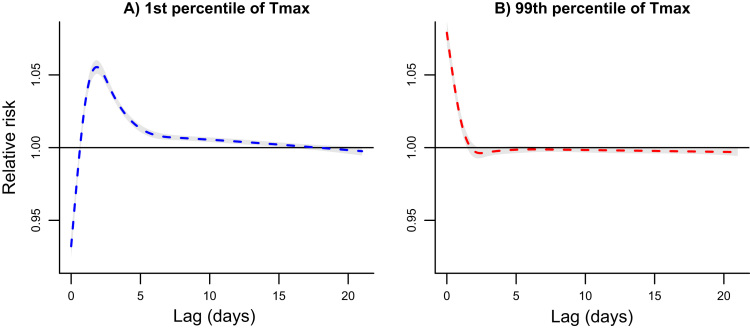
Pooled relative risk of all-age all-cause mortality over the 21-day lag period at the 1st (panel A) and 99th (panel B) percentile of the distribution of daily maximum temperatures (Tmax) relative to the minimum mortality temperature.

The timing of risk differed for exposure to cold and heat. The cold effect peaked on the second or third day after exposure and persisted at a low level for two weeks or more (Fig. 4). In contrast, the strongest heat effects occurred immediately but diminished quickly, followed by an extended period of slightly negative risk that may suggest (marginal) mortality displacement (Fig. 4).

Changing modeling assumptions generally had a modest impact on results, with attributable fractions remaining between 2.5% and 5.0% (Table S3). Of the parameters tested, the length of the lag had the largest influence, with a shorter lag reducing the size of the effect and vice versa.

## 4 Discussion

This is the first long-term, national-level study of temperature-mortality associations in South Africa or anywhere on the African continent. Our findings provide evidence of an increased risk of death from both cold and heat, and suggest that approximately 3.4% of the total mortality burden in the country was related to non-optimum temperatures over the 17-year study period. The majority of the burden (almost 90%) was from cold, defined as days with temperatures below the optimum, and most was from moderate rather than extreme cold, which is in line with previous research (Gasparrini et al., 2015b).

A total attributable mortality of 3.4% is at the lower end of the levels recently reported in a 13-country analysis using related methods (Gasparrini et al., 2015b). In that study, the two countries (Brazil and Thailand) with the most similar overall attributable mortality were also two of the most similar to South Africa in national-level indicators of economic development, such as GDP per capita and income inequality (World Bank, 2017). How these factors relate to temperature vulnerability may warrant additional study.

We found a MMP of 84, which is higher than what is often reported in multi-country studies (Guo et al., 2014). However, we use the daily maximum rather than daily mean temperature, making direct comparisons difficult. In terms of lag-response relationships, our finding of a relatively immediate and short-lived heat effect, but a delayed and persistent cold effect is commonly reported (Braga et al., 2002, Guo et al., 2014).

In sub-group analyses, we found evidence of elevated risks from cardiovascular and respiratory diseases and in the oldest and youngest age groups. Though not universal, these results are consistent with studies from around the world, including in parts of Africa (Basu, 2009, Benmarhnia et al., 2015, Diboulo et al., 2012, Egondi et al., 2012, Ryti et al., 2016, Wichmann, 2017). Higher risks amongst the elderly and for cardiorespiratory causes may have important implications for how the temperature-mortality association in South Africa may evolve over time as the population continues to age and mortality further transitions away from infectious causes and towards chronic disease (Mayosi et al., 2009, United Nations, 2015).

The ability to investigate temperature effects in children < 5 years is a particular strength of the study, as there are relatively few papers reporting results in such young age groups. We found evidence of a heat- and cold-effect, though the former was more definitive. Heat vulnerability in young children has been reported in systematic reviews and several individual studies in developing regions (Azongo et al., 2012, Basu, 2009, Diboulo et al., 2012, Geruso and Spears, 2017, Xu et al., 2012).

There is only one previously published time-series study on temperature-related mortality in South Africa, which analyzes data from Cape Town between 1996 and 1999. Similar to our study, they reported evidence of cold-related mortality but no clear heat effect in Cape Town (McMichael et al., 2008). A similar result was also found in an unpublished time-series analysis of Cape Town using newer data (2001–2004) (Kovats, 2010). In contrast, a recent case-crossover study based on data from 2006 to 2010 did find an association between all-age all-cause mortality and heat in Cape Town, but using an alternative temperature metric (apparent temperature) and different analytical method (Wichmann, 2017). Like our results, that study did not report significant associations in the other two cities investigated (City of Johannesburg and Durban). All four studies relied on the same source of mortality data, albeit for different (but partially overlapping) time periods.

The mortality dataset itself is a potential source of error, as Statistics South Africa estimates that up to 11% of deaths may not have been captured by the national registry early in our study period, declining to around 6% by the end (Statistics South Africa, Mortality and causes of death in South Africa, 2011, Statistics South Africa, Mortality and causes of death in South Africa, 2013). External mortality estimates suggest the incompleteness may be larger (Institute for Health Metrics and Evaluation, 2015). If missing death records are random, they would likely have little impact on our relative risk estimates, but if exclusions were somehow systematically related to temperature, they could be meaningful.

The temperature dataset also posed certain challenges and limitations. The most obvious is that we did not have data for all days in all districts, with much of the missing data concentrated in the early periods. Specifically, 11% of deaths had no corresponding temperature data, although this varied across districts. For many districts and time periods however, we were able to obtain data from multiple stations, helping us conduct a quality control procedure that was based in part on comparisons with nearby locations. This process allowed us to select the best available dataset for each district and to exclude problematic data points (duplicates and false outliers). Ultimately, we selected the maximum daily temperature as our metric of choice, instead of the minimum or mean, because the minimums had substantially more missing data (12% vs 7%). Previous analyses indicate that, on average, different temperature measures have similar predictive abilities, due largely to their strong correlation, and therefore practical concerns like data completeness should be the determining factor (Barnett et al., 2010, Basu et al., 2008). A final limitation with regard to temperature is that for some larger districts, the use of a single station may not adequately capture all local weather conditions, though this issue was somewhat tempered by the fact that total mortality was more concentrated in the smaller districts; for example, only 14% of deaths occurred in the largest 25% of districts.

Some researchers investigating temperature-mortality relationships include ambient air pollution as a potential confounder *a priori*, since it can be correlated with temperature, while others argue that such control is inappropriate because air pollution is on the causal pathway (McMichael et al., 2008). We did not have air pollution data, but note that large-scale studies exploring the issue generally find that it has only a small influence on effect estimates and may even strengthen the temperature association (Basu et al., 2008, Gasparrini et al., 2015b, McMichael et al., 2008). Accordingly, a previous study from Cape Town reported that controlling for ozone had a very small impact overall while controlling for particulate air pollution increased the heat effect (McMichael et al., 2008).

In conclusion, our work provides valuable insights into temperature-mortality relationships in a country and region that is understudied and underrepresented in the literature. The results can help inform early warning systems and heat-health action plans and be used to project future mortality impacts under climate change.
