# Supplementary material for: The association between ambient temperature and mortality in South Africa: A time-series analysis
Source: Environ Res. 2018 Feb;161:229–35. doi: 10.1016/j.envres.2017.11.001 (PMC5773242; doi:10.1016/j.envres.2017.11.001)
Supplement: Supplementary file 1 — Supplementary material [file mmc1.docx]

**Supplementary Material**

**The association between ambient temperature and mortality in South Africa: a time-series analysis**

**Table of Contents**

| **Figure S1.** Number of districts (n=52) with temperature data over the study period. |
| --- |
|  |
| **Figure S2**. Best-linear unbiased predictions of the overall cumulative exposure response associations for all 52 districts in South Africa. |
| **Table S1**. Deaths (all-age, all-cause), average daily maximum temperature (Temp), minimum mortality temperatures and percentiles (MMTs and MMPs), relative risks at select temperatures and attributable fractions from heat and cold.  **Table S2.** Attributable mortality fraction from cold and heat by district, computed for “extreme” temperature ranges below and above the 2.5^th^ and 97.5^th^ temperature percentiles, respectively. |
| **Table S3.** Results of sensitivity analysis of the modeling choices, reported as attributable fractions. |
|  |
|  |
|  |


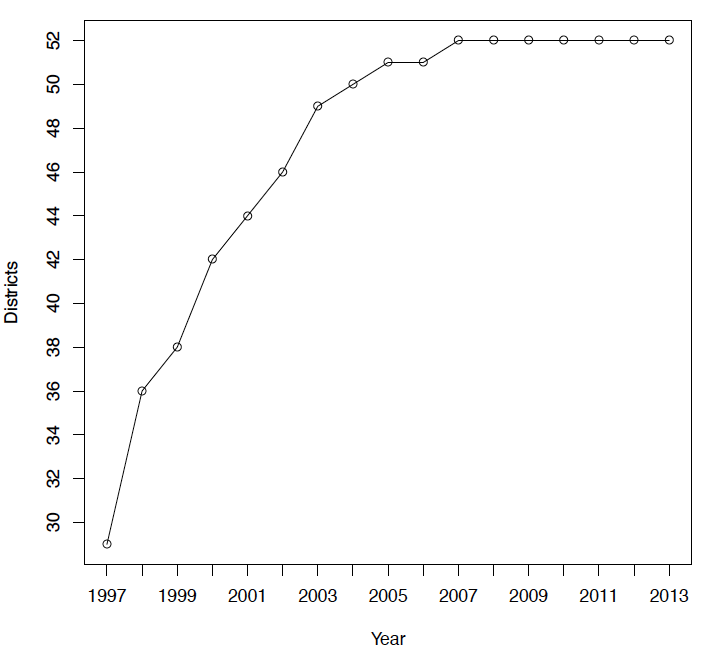


**Figure S1.** Number of districts (n=52) with temperature data over the study period.

**Figure S2**. Best-linear unbiased predictions of the overall cumulative exposure response associations for all 52 districts in South Africa. The light grey vertical line shows the minimum mortality temperature and the darker grey vertical lines show the 2.5^th^ and 97.5^th^ temperature percentiles. The secondary y-axis (and associated histogram) shows the attributable deaths at each temperature.

**Table S1**. Deaths (all-age, all-cause), average daily maximum temperature (Temp), minimum mortality temperatures and percentiles (MMTs and MMPs), relative risks at select temperatures and total attributable fractions and deaths from heat and cold.

|  | **Province^a^** | **Deaths** | | **Temp** | **MMP** | **MMT** | **Relative risk** | | **Attributable fraction (%)** | | **Attributable deaths^d^** | |
| --- | --- | --- | --- | --- | --- | --- | --- | --- | --- | --- | --- | --- |
|  |  | **Total^b^** | **Included^c^** |  |  |  | **1^st^ %tile** | **99^th^ %tile** | **Cold** | **Heat** | **Cold** | **Heat** |
| Alfred Nzo | EC | 114,467 | 94,731 | 21.6 | 91 | 28.8 | 1.20 | 1.10 | 4.2 | 0.3 | 4,799 | 326 |
| Amajuba | KZN | 95,209 | 50,282 | 25.7 | 55 | 26.9 | 1.24 | 1.14 | 2.9 | 1.5 | 2,734 | 1,448 |
| Amathole | EC | 235,000 | 229,182 | 24.7 | 72 | 28.2 | 1.23 | 1.14 | 3.1 | 0.7 | 7,266 | 1,644 |
| Bojanala | NW | 215,910 | 183,444 | 26.5 | 5 | 18.5 | 1.01 | 1.21 | 0.0 | 3.5 | 42 | 7,522 |
| Buffalo City | EC | 183,536 | 181,276 | 15.8 | 93 | 21.0 | 1.18 | 1.06 | 6.0 | 0.2 | 10,947 | 299 |
| Cacadu | EC | 76,341 | 70,821 | 25.5 | 90 | 34.1 | 1.21 | 1.11 | 3.0 | 0.3 | 2,326 | 252 |
| Cape Winelands | WC | 101,671 | 100,689 | 22.8 | 60 | 25.5 | 1.25 | 1.29 | 2.7 | 1.1 | 2,754 | 1,149 |
| Capricorn | LIM | 205,787 | 197,313 | 26.8 | 99 | 35.0 | 1.07 | 1.00 | 3.2 | 0.0 | 6,671 | -23 |
| Central Karoo | WC | 13,873 | 13,754 | 25.7 | 84 | 32.8 | 1.16 | 1.16 | 2.0 | 0.6 | 279 | 83 |
| Chris Hani | EC | 141,243 | 134,875 | 23.4 | 88 | 31.4 | 1.20 | 1.12 | 3.4 | 0.4 | 4,735 | 605 |
| City of Cape Town | WC | 447,086 | 444,753 | 23.7 | 99 | 35.6 | 1.21 | 1.00 | 6.0 | 0.0 | 26,950 | -10 |
| City of Johannesburg | GT | 553,340 | 538,957 | 23.2 | 99 | 31.1 | 1.23 | 1.00 | 6.0 | 0.0 | 33,097 | -102 |
| City of Tshwane | GP | 370,121 | 360,724 | 24.4 | 84 | 28.8 | 1.10 | 1.07 | 1.0 | 0.3 | 3,613 | 1,023 |
| Dr Kenneth Kaunda | NW | 156,481 | 146,114 | 27.2 | 83 | 32.0 | 1.05 | 1.06 | 0.9 | 0.2 | 1,412 | 324 |
| Dr Ruth Segomotsi Mompati | NW | 84,956 | 75,323 | 29.1 | 52 | 30.1 | 1.26 | 1.20 | 3.3 | 2.5 | 2,799 | 2,086 |
| Eden | WC | 82,205 | 81,635 | 24.3 | 79 | 30.1 | 1.24 | 1.17 | 2.6 | 0.7 | 2,155 | 572 |
| Ehlanzeni | MP | 258,712 | 216,835 | 27.1 | 89 | 32.6 | 1.06 | 1.01 | 1.0 | 0.0 | 2,674 | 111 |
| Ekurhuleni | GT | 449,732 | 338,655 | 23.4 | 90 | 28.9 | 1.10 | 1.03 | 3.2 | 0.1 | 14,199 | 388 |
| eThekwini | KZN | 582,400 | 578,713 | 26.6 | 99 | 33.5 | 1.13 | 1.00 | 2.8 | 0.0 | 16,226 | -130 |
| Fezile Dabi | FS | 106,758 | 84,913 | 25.3 | 82 | 30.3 | 1.13 | 1.08 | 1.3 | 0.3 | 1,394 | 343 |
| Frances Baard | NC | 80,748 | 79,737 | 27.6 | 56 | 29.4 | 1.18 | 1.09 | 2.5 | 1.0 | 1,984 | 772 |
| Gert Sibande | MP | 201,085 | 168,584 | 22.8 | 92 | 29.0 | 1.13 | 1.04 | 4.1 | 0.1 | 8,200 | 251 |
| Greater Sekhukhune | LIM | 154,245 | 108,751 | 28.4 | 51 | 29.0 | 1.09 | 1.06 | 0.9 | 0.4 | 1,326 | 655 |
| iLembe | KZN | 96,687 | 59,964 | 26.6 | 26 | 23.8 | 1.09 | 1.11 | 0.5 | 0.8 | 442 | 797 |
| Joe Gqabi | EC | 70,534 | 48,732 | 24.9 | 90 | 32.3 | 1.14 | 1.05 | 3.8 | 0.2 | 2,681 | 123 |
| John Taolo Gaetsewe | NC | 38,604 | 17,535 | 27.9 | 55 | 29.5 | 1.26 | 1.11 | 3.5 | 1.5 | 1,335 | 565 |
| Lejweleputswa | FS | 172,065 | 139,364 | 26.0 | 89 | 32.7 | 1.14 | 1.05 | 2.5 | 0.2 | 4,377 | 300 |
| Mangaung | FS | 186,482 | 184,144 | 26.1 | 61 | 28.5 | 1.21 | 1.07 | 3.7 | 0.8 | 6,860 | 1,404 |
| Mopani | LIM | 154,962 | 123,881 | 29.0 | 53 | 29.4 | 1.07 | 1.16 | 0.9 | 1.3 | 1,452 | 2,000 |
| Namakwa | NC | 16,347 | 16,228 | 25.5 | 51 | 26.2 | 1.21 | 1.20 | 1.8 | 1.1 | 295 | 177 |
| Nelson Mandela Bay | EC | 215,125 | 213,805 | 23.8 | 95 | 30.4 | 1.06 | 1.02 | 2.6 | 0.1 | 5,594 | 161 |
| Ngaka Modiri Molema | NW | 158,209 | 156,926 | 27.5 | 56 | 28.9 | 1.12 | 1.05 | 1.9 | 0.7 | 3,006 | 1,145 |
| Nkangala | MP | 195,810 | 147,481 | 24.8 | 92 | 30.8 | 1.03 | 1.05 | 4.0 | 0.2 | 7,864 | 320 |
| O.R.Tambo | EC | 194,455 | 181,489 | 24.0 | 89 | 30.9 | 1.16 | 1.10 | 2.7 | 0.4 | 5,163 | 730 |
| Overberg | WC | 30,406 | 30,243 | 23.4 | 89 | 30.0 | 1.22 | 1.06 | 3.7 | 0.2 | 1,129 | 58 |
| Pixley ka Seme | NC | 42,781 | 42,449 | 26.2 | 83 | 33.2 | 1.20 | 1.05 | 1.9 | 0.2 | 829 | 103 |
| Sedibeng | GT | 174,249 | 95,186 | 25.4 | 57 | 26.9 | 1.17 | 1.03 | 1.6 | 0.2 | 2,836 | 414 |
| Sisonke | KZN | 92,073 | 85,597 | 21.4 | 88 | 27.3 | 1.24 | 1.04 | 3.9 | 0.2 | 3,622 | 168 |
| Siyanda | NC | 47,034 | 46,271 | 30.0 | 50 | 30.7 | 1.19 | 1.09 | 2.0 | 1.1 | 921 | 509 |
| Thabo Mofutsanyane | FS | 196,340 | 195,601 | 23.4 | 98 | 31.1 | 1.21 | 1.00 | 5.1 | 0.0 | 10,102 | 11 |
| Ugu | KZN | 169,571 | 158,976 | 24.9 | 99 | 32.5 | 1.12 | 1.00 | 3.2 | 0.0 | 5,451 | -17 |
| uMgungundlovu | KZN | 227,558 | 223,668 | 19.7 | 89 | 25.9 | 1.26 | 1.08 | 4.3 | 0.3 | 9,899 | 637 |
| uMkhanyakude | KZN | 92,434 | 75,570 | 27.8 | 52 | 28.0 | 1.10 | 1.05 | 0.8 | 0.3 | 719 | 240 |
| uMzinyathi | KZN | 106,810 | 103,995 | 22.7 | 89 | 29.2 | 1.21 | 1.05 | 3.4 | 0.2 | 3,633 | 198 |
| uThukela | KZN | 140,953 | 127,948 | 22.7 | 91 | 28.5 | 1.10 | 1.04 | 4.4 | 0.1 | 6,203 | 199 |
| uThungulu | KZN | 182,682 | 167,855 | 29.4 | 99 | 39.0 | 1.12 | 1.00 | 3.5 | 0.0 | 6,450 | -56 |
| Vhembe | LIM | 134,651 | 82,998 | 30.6 | 57 | 31.5 | 1.01 | 1.13 | 0.8 | 1.4 | 1,011 | 1,849 |
| Waterberg | LIM | 74,795 | 60,366 | 29.4 | 40 | 28.2 | 1.08 | 1.07 | 0.6 | 0.7 | 450 | 535 |
| West Coast | WC | 58,114 | 57,769 | 26.1 | 58 | 28.1 | 1.27 | 1.17 | 3.8 | 1.1 | 2,214 | 631 |
| West Rand | GT | 160,416 | 100,396 | 24.1 | 61 | 25.9 | 1.16 | 1.07 | 1.5 | 0.4 | 2,362 | 626 |
| Xhariep | FS | 50,127 | 44,861 | 27.0 | 89 | 35.0 | 1.22 | 1.05 | 2.2 | 0.2 | 1,118 | 93 |
| Zululand | KZN | 117,950 | 107,315 | 25.9 | 89 | 31.4 | 1.09 | 1.05 | 2.0 | 0.2 | 2,411 | 198 |
| ^a^ EC = Eastern Cape, FS = Free State, GT = Gauteng, KZN = KwaZulu-Natal, LIM = Limpopo, MP = Mpumalanga, NC = Northern Cape, NW = Northwest, WC = Western Cape.  ^b^ Excludes stillbirths, deaths without location information and nonsensical dates of death.  ^c^ Deaths were included for all days with temperature data.  ^d^ Based on total deaths. | | | | | | | | | | | | |

**Table S2**. Attributable mortality fraction from cold and heat by district, computed for “extreme” temperature ranges below and above the 2.5^th^ and 97.5^th^ temperature percentiles, respectively.

|  |  | **t ≤ 2.5th** | **t ≥ 97.5** |
| --- | --- | --- | --- |
| Alfred Nzo | Cold (%) | 0.44 (0.16,0.70) | 0.00 (0.00,0.00) |
|  | Heat (%) | 0.00 (0.00,0.00) | 0.20 (-0.06,0.46) |
| Amajuba | Cold (%) | 0.51 (0.22,0.76) | 0.00 (0.00,0.00) |
|  | Heat (%) | 0.00 (0.00,0.00) | 0.29 (0.00,0.57) |
| Amathole | Cold (%) | 0.50 (0.25,0.70) | 0.00 (0.00,0.00) |
|  | Heat (%) | 0.00 (0.00,0.00) | 0.30 (0.00,0.61) |
| Bojanala | Cold (%) | 0.02 (-0.13,0.14) | 0.00 (0.00,0.00) |
|  | Heat (%) | 0.00 (0.00,0.00) | 0.43 (0.15,0.75) |
| Buffalo City | Cold (%) | 0.39 (0.01,0.68) | 0.00 (0.00,0.00) |
|  | Heat (%) | 0.00 (0.00,0.00) | 0.14 (-0.25,0.47) |
| Cacadu | Cold (%) | 0.48 (0.16,0.79) | 0.00 (0.00,0.00) |
|  | Heat (%) | 0.00 (0.00,0.00) | 0.22 (-0.06,0.51) |
| Cape Winelands | Cold (%) | 0.57 (0.27,0.82) | 0.00 (0.00,0.00) |
|  | Heat (%) | 0.00 (0.00,0.00) | 0.51 (0.29,0.87) |
| Capricorn | Cold (%) | 0.17 (-0.23,0.54) | 0.01 (-0.02,0.04) |
|  | Heat (%) | 0.00 (0.00,0.00) | -0.01 (-0.06,0.03) |
| Central Karoo | Cold (%) | 0.37 (0.01,0.72) | 0.00 (0.00,0.00) |
|  | Heat (%) | 0.00 (0.00,0.00) | 0.32 (0.04,0.69) |
| Chris Hani | Cold (%) | 0.46 (0.18,0.71) | 0.00 (0.00,0.00) |
|  | Heat (%) | 0.00 (0.00,0.00) | 0.28 (0.06,0.51) |
| City of Cape Town | Cold (%) | 0.48 (0.25,0.70) | 0.00 (-0.02,0.03) |
|  | Heat (%) | 0.00 (0.00,0.00) | 0 (-0.02,0.02) |
| City of Johannesburg | Cold (%) | 0.51 (0.28,0.70) | 0.02 (-0.01,0.05) |
|  | Heat (%) | 0.00 (0.00,0.00) | -0.02 (-0.05,0.00) |
| City of Tshwane | Cold (%) | 0.24 (0.09,0.37) | 0.00 (0.00,0.00) |
|  | Heat (%) | 0.00 (0.00,0.00) | 0.17 (-0.03,0.36) |
| Dr Kenneth Kaunda | Cold (%) | 0.13 (-0.13,0.35) | 0.00 (0.00,0.00) |
|  | Heat (%) | 0.00 (0.00,0.00) | 0.12 (-0.05,0.29) |
| Dr Ruth Segomotsi Mompati | Cold (%) | 0.55 (0.26,0.82) | 0.00 (0.00,0.00) |
|  | Heat (%) | 0.00 (0.00,0.00) | 0.44 (0.18,0.71) |
| Eden | Cold (%) | 0.52 (0.20,0.77) | 0.00 (0.00,0.00) |
|  | Heat (%) | 0.00 (0.00,0.00) | 0.34 (0.09,0.61) |
| Ehlanzeni | Cold (%) | 0.14 (-0.15,0.37) | 0.00 (0.00,0.00) |
|  | Heat (%) | 0.00 (0.00,0.00) | 0.03 (-0.20,0.22) |
| Ekurhuleni | Cold (%) | 0.26 (0.06,0.44) | 0.00 (0.00,0.00) |
|  | Heat (%) | 0.00 (0.00,0.00) | 0.06 (-0.08,0.21) |
| eThekwini | Cold (%) | 0.28 (0.02,0.51) | 0.01 (-0.01,0.02) |
|  | Heat (%) | 0.00 (0.00,0.00) | -0.02 (-0.07,0.01) |
| Fezile Dabi | Cold (%) | 0.31 (0.06,0.48) | 0.00 (0.00,0.00) |
|  | Heat (%) | 0.00 (0.00,0.00) | 0.17 (-0.06,0.41) |
| Frances Baard | Cold (%) | 0.41 (0.11,0.69) | 0.00 (0.00,0.00) |
|  | Heat (%) | 0.00 (0.00,0.00) | 0.20 (-0.08,0.50) |
| Gert Sibande | Cold (%) | 0.30 (0.03,0.53) | 0.00 (0.00,0.00) |
|  | Heat (%) | 0.00 (0.00,0.00) | 0.10 (-0.08,0.28) |
| Greater Sekhukhune | Cold (%) | 0.22 (-0.08,0.48) | 0.00 (0.00,0.00) |
|  | Heat (%) | 0.00 (0.00,0.00) | 0.14 (-0.08,0.38) |
| iLembe | Cold (%) | 0.20 (-0.05,0.38) | 0.00 (0.00,0.00) |
|  | Heat (%) | 0.00 (0.00,0.00) | 0.27 (-0.10,0.68) |
| Joe Gqabi | Cold (%) | 0.31 (-0.01,0.57) | 0.00 (0.00,0.00) |
|  | Heat (%) | 0.00 (0.00,0.00) | 0.11 (-0.10,0.33) |
| John Taolo Gaetsewe | Cold (%) | 0.55 (0.21,0.88) | 0.00 (0.00,0.00) |
|  | Heat (%) | 0.00 (0.00,0.00) | 0.22 (-0.15,0.59) |
| Lejweleputswa | Cold (%) | 0.32 (0.03,0.57) | 0.00 (0.00,0.00) |
|  | Heat (%) | 0.00 (0.00,0.00) | 0.12 (-0.01,0.25) |
| Mangaung | Cold (%) | 0.48 (0.23,0.70) | 0.00 (0.00,0.00) |
|  | Heat (%) | 0.00 (0.00,0.00) | 0.13 (-0.04,0.31) |
| Mopani | Cold (%) | 0.17 (-0.07,0.40) | 0.00 (0.00,0.00) |
|  | Heat (%) | 0.00 (0.00,0.00) | 0.35 (0.10,0.66) |
| Namakwa | Cold (%) | 0.50 (0.07,0.91) | 0.00 (0.00,0.00) |
|  | Heat (%) | 0.00 (0.00,0.00) | 0.43 (0.00,0.85) |
| Nelson Mandela Bay | Cold (%) | 0.14 (-0.11,0.36) | 0.00 (0.00,0.00) |
|  | Heat (%) | 0.00 (0.00,0.00) | 0.07 (-0.10,0.24) |
| Ngaka Modiri Molema | Cold (%) | 0.30 (-0.01,0.58) | 0.00 (0.00,0.00) |
|  | Heat (%) | 0.00 (0.00,0.00) | 0.12 (-0.10,0.35) |
| Nkangala | Cold (%) | 0.08 (-0.16,0.30) | 0.00 (0.00,0.00) |
|  | Heat (%) | 0.00 (0.00,0.00) | 0.13 (-0.09,0.33) |
| O.R.Tambo | Cold (%) | 0.37 (0.14,0.56) | 0.00 (0.00,0.00) |
|  | Heat (%) | 0.00 (0.00,0.00) | 0.25 (0.01,0.50) |
| Overberg | Cold (%) | 0.52 (0.21,0.79) | 0.00 (0.00,0.00) |
|  | Heat (%) | 0.00 (0.00,0.00) | 0.15 (-0.09,0.38) |
| Pixley ka Seme | Cold (%) | 0.49 (0.18,0.78) | 0.00 (0.00,0.00) |
|  | Heat (%) | 0.00 (0.00,0.00) | 0.12 (-0.12,0.36) |
| Sedibeng | Cold (%) | 0.42 (0.13,0.68) | 0.00 (0.00,0.00) |
|  | Heat (%) | 0.00 (0.00,0.00) | 0.06 (-0.25,0.35) |
| Sisonke | Cold (%) | 0.48 (0.27,0.66) | 0.00 (0.00,0.00) |
|  | Heat (%) | 0.00 (0.00,0.00) | 0.11 (-0.19,0.41) |
| Siyanda | Cold (%) | 0.44 (0.06,0.75) | 0.00 (0.00,0.00) |
|  | Heat (%) | 0.00 (0.00,0.00) | 0.21 (-0.21,0.55) |
| Thabo Mofutsanyane | Cold (%) | 0.48 (0.25,0.71) | 0.00 (0.00,0.00) |
|  | Heat (%) | 0.00 (0.00,0.00) | 0.01 (-0.04,0.05) |
| Ugu | Cold (%) | 0.28 (0.00,0.51) | 0.01 (-0.02,0.04) |
|  | Heat (%) | 0.00 (0.00,0.00) | -0.01 (-0.06,0.03) |
| uMgungundlovu | Cold (%) | 0.52 (0.29,0.69) | 0.00 (0.00,0.00) |
|  | Heat (%) | 0.00 (0.00,0.00) | 0.19 (-0.03,0.44) |
| uMkhanyakude | Cold (%) | 0.24 (-0.08,0.50) | 0.00 (0.00,0.00) |
|  | Heat (%) | 0.00 (0.00,0.00) | 0.14 (-0.1,0.42) |
| uMzinyathi | Cold (%) | 0.43 (0.19,0.59) | 0.00 (0.00,0.00) |
|  | Heat (%) | 0.00 (0.00,0.00) | 0.13 (-0.12,0.37) |
| uThukela | Cold (%) | 0.25 (0.07,0.42) | 0.00 (0.00,0.00) |
|  | Heat (%) | 0.00 (0.00,0.00) | 0.12 (-0.07,0.29) |
| uThungulu | Cold (%) | 0.27 (-0.19,0.62) | 0.02 (-0.02,0.06) |
|  | Heat (%) | 0.00 (0.00,0.00) | -0.03 (-0.10,0.04) |
| Vhembe | Cold (%) | 0.03 (-0.36,0.38) | 0.00 (0.00,0.00) |
|  | Heat (%) | 0.00 (0.00,0.00) | 0.28 (-0.08,0.69) |
| Waterberg | Cold (%) | 0.18 (-0.12,0.51) | 0.00 (0.00,0.00) |
|  | Heat (%) | 0.00 (0.00,0.00) | 0.16 (-0.02,0.37) |
| West Coast | Cold (%) | 0.58 (0.26,0.86) | 0.00 (0.00,0.00) |
|  | Heat (%) | 0.00 (0.00,0.00) | 0.34 (0.10,0.70) |
| West Rand | Cold (%) | 0.37 (0.11,0.58) | 0.00 (0.00,0.00) |
|  | Heat (%) | 0.00 (0.00,0.00) | 0.16 (-0.11,0.44) |
| Xhariep | Cold (%) | 0.49 (0.09,0.81) | 0.00 (0.00,0.00) |
|  | Heat (%) | 0.00 (0.00,0.00) | 0.14 (0.00,0.28) |
| Zululand | Cold (%) | 0.21 (0.00,0.41) | 0.00 (0.00,0.00) |
|  | Heat (%) | 0.00 (0.00,0.00) | 0.12 (-0.08,0.33) |
| Total | Cold (%) | 0.33 (0.28,0.37) | 0 (0.00,0.01) |
|  | Heat (%) | 0.00 (0.00,0.00) | 0.13 (0.11,0.17) |

**Table S3.** Results of sensitivity analysis of the modeling choices, reported as attributable fractions. Values are rounded.

| **Modeling choices** | **Total (%)** | **Cold (%)** | **Heat (%)** |
| --- | --- | --- | --- |
| Main model | 3.4 | 3.0 | 0.4 |
| Knots for exposure-response: 10^th^, 25^th^, 75^th^, 90^th^ | 3.8 | 3.1 | 0.7 |
| Quadratic b-spline for exposure response | 3.8 | 3.3 | 0.5 |
| Df for lag response: 6 | 3.3 | 2.8 | 0.4 |
| Lag of 14 days | 2.5 | 2.0 | 0.5 |
| Lag of 28 days | 5.0 | 4.4 | 0.6 |
| Df/season control: 6 | 3.4 | 2.9 | 0.4 |
| Df/season control: 10 | 3.9 | 3.6 | 0.3 |
